# Optimizing hyperbaric oxygen therapy for PTSD—The importance of dose and duration for sustained benefits

**DOI:** 10.3389/fneur.2024.1447742

**Published:** 2024-09-26

**Authors:** Keren Doenyas-Barak, Shai Efrati

**Affiliations:** ^1^Yitzhak Shamir Medical Center, Tel Aviv, Israel; ^2^The Sagol Center for Hyperbaric Medicine and Research, Assaf Harofeh Medical Center, Ramle, Israel; ^3^School of Medicine, Sackler Faculty of Medicine, Tel Aviv University, Tel Aviv, Israel

**Keywords:** hyperbaric oxygen therapy (HBOT), posttraumatic stress disorder (PTSD), treatment response, memory, treatment dose

In their manuscript “Systematic Review and Dosage Analysis: Hyperbaric Oxygen Therapy Efficacy in the Treatment of Post-Traumatic Stress Disorder,” (1). Andrews and Harch describe a dose-response curve of hyperbaric oxygen therapy on the post-traumatic symptoms of patients with PTSD. The authors calculated “atmosphere-minutes” (AMs) given in each protocol using a formula that multiplies the atmospheric pressure, fraction of oxygen, session length, and number of sessions. They have found a linear dose-response relationship, with increased symptomatic improvement corresponding to increasing cumulative oxygen doses from 1,002 to 11,400 atmosphere-minutes of oxygen. For example, while the mean decrease in post-traumatic symptom load was 17% to 18% when prescribing 3,000 to 7,000 AMs, it was 30% at 11,400 AMs.

The authors then state that the greater symptomatic response was accompanied by a reversible exacerbation of emotional symptoms at the highest oxygen doses in 30%−39% of subjects. They offer considering sticking to lower doses to avoid emotional exacerbation. However, clinical practice suggests otherwise.

And indeed, the so called “symptom worsening,” frequently accompanied by recollection of previously inaccessible memories, is very common during intensive protocols, necessitating careful and experienced support. While the authors regard memory recollection and the accompanying distress as adverse effects, it may represent an “on-target” effect associated with the direct impact of hyperbaric oxygen therapy (HBOT) on hippocampal-based memory processing in individuals with PTSD (2, 3).

The hippocampus may be more sensitive to HBOT than any other brain region. Studies on hippocampal cell cultures have shown that fluctuations in both pressure and oxygen levels can directly induce orthodromic activity and neural plasticity (4). An increase in hypoxia-inducible factor (HIF) levels related to HBOT has also been shown to contribute to improved hippocampal functions and memory performance (5–7). Furthermore, improved hippocampus activity and connectivity demonstrated in brain imaging following HBOT among veterans with PTSD also support this notion (8).

However, for neuroplasticity to be induced and for long-lasting rather than temporary improvement of symptoms to occur, a long treatment course may be needed. Thus, while short-term response to HBOT using lower AMs may be fair, it is the long-term effect and improvement persistence that may better reflect the structural effect of the different protocols.

Most of the studies in the Andrews and Harch manuscript that evaluated the effect of HBOT on PTSD assessed the short-term effects after treatment completion (Table 1). Only three of the studies also evaluated long-term effects at intervals of 6 months to 2 years. Weaver et al. (9) provided 3,120 AMs and reported a 17% decrease immediately after completion of the HBOT treatment protocol, and attenuation of the improvement to 7% after 6 months and a 10% worsening of symptoms at 12 months. In contrast, in Harch et al. (10), an intensive protocol with twice-daily sessions and 3,420 AMs was prescribed, reporting a 26% decrease in symptoms evaluated immediately after the treatment course completion and “further improvement” at the 6-month evaluation. But Harch also reported transient worsening in some symptoms among 7 of the 30 study participants (of whom 23 had PTSD). It is not clear if the symptom worsening was occurred in the post-traumatic population.

**Table 1:** Time of evaluation and AMs related treatment effect.

**Studies referenced in Andrews and Harch**	**AMs**	**Time of evaluation following intervention**	**Change in post-traumatic score**
Wolf et al. (13)	6,900	6 Weeks	−17%
Cifu et al. (12)	4,860	Immediately	−14%
Harch et al. (10)	3,420	Immediately	−26%
		6 Months	“Further improvement”
Miller et al. (14)	3,120	Immediately	−10%
Weaver et al. (9)	3,120	Immediately	−17%
		6 Months	−7%
		12 Months	+10%
Hadanny et al. (15)	11,400	Immediately	−28%
Doenyas-Barak et al. (8, 11)	11,400	Immediately	−39%
		700 days	−44%

In Doenyas-Barak et al. (11), the short term improvement was 39% and the improvement persisted together with improved social and occupational function 2 years after completion of the 11,400 AMs protocol. Cifu et al. (12) stated in their manuscript that a second evaluation of 3-month time periods following HBOT would be reported; however, no such publication could be found.

To conclude, higher AMs may be necessary not just for better short-term response, but also for changes in brain activity and memory perception that may contribute to long-lasting effects. In addition, given the almost linear correlation between dose and response, the question is whether longer courses are needed to achieve even better respond.
